# Identifying Coronary Artery Disease in Asymptomatic Middle-Aged Sportsmen: The Additional Value of Pulse Wave Velocity

**DOI:** 10.1371/journal.pone.0131895

**Published:** 2015-07-06

**Authors:** Thijs L. Braber, Niek H. J. Prakken, Arend Mosterd, Willem P. Th. M. Mali, Pieter A. F. M. Doevendans, Michiel L. Bots, Birgitta K. Velthuis

**Affiliations:** 1 Department of Radiology, University Medical Center Utrecht, The Netherlands; 2 Department of Cardiology, University Medical Center Utrecht, The Netherlands; 3 Department of Radiology, University Medical Center Groningen, The Netherlands; 4 Department of Cardiology, Meander Medical Center, Amersfoort, The Netherlands; 5 Julius Center for Health Sciences and Primary Care, University Medical Center Utrecht, The Netherlands; Tel Aviv Sourasky Medical Center, ISRAEL

## Abstract

**Background:**

Cardiovascular screening may benefit middle-aged sportsmen, as coronary artery disease (CAD) is the main cause of exercise-related sudden cardiac death. Arterial stiffness, as measured by pulse wave velocity (PWV), may help identify sportsmen with subclinical CAD. We examined the additional value of PWV measurements to traditional CAD risk factors for identifying CAD.

**Methods:**

From the Measuring Athlete’s Risk of Cardiovascular events (MARC) cohort of asymptomatic, middle-aged sportsmen who underwent low-dose Cardiac CT (CCT) after routine sports medical examination (SME), 193 consecutive sportsmen (aged 55±6.6 years) were included with additional PWV measurements before CCT. Sensitivity, specificity and predictive values of PWV values (>8.3 and >7.5m/s) assessed by Arteriograph were used to identify CAD (coronary artery calcium scoring ≥100 Agatston Units or coronary CT angiography luminal stenosis ≥50%) and to assess the additional diagnostic value of PWV to established cardiovascular risk factors.

**Results:**

Forty-seven sportsmen (24%) had CAD on CCT. They were older (58.9 vs. 53.8 years, p<0.001), had more hypertension (17 vs. 4%, p=0.003), higher cholesterol levels (5.7 vs. 5.4mmol/l) p=0.048), and more often were (ever) smokers (55 vs. 34%, p=0.008). Mean PWV was higher in those with CAD (8.9 vs. 8.0 m/s, p=0.017). For PWV >8.3m/s respectively >7.5m/s sensitivity to detect CAD on CT was 43% and 74%, specificity 69% and 45%, positive predictive value 31% and 30%, and negative predictive value 79% and 84%. Adding PWV to traditional risk factor models did not change the area under the curve (from 0.78 (95% CI = 0.709-0.848)) to AUC 0.78 (95% CI 0.710-0.848, p = 0.99)) for prediction of CAD on CCT.

**Conclusions:**

Limited additional value was found for PWV on top of established risk factors to identify CAD. PWV might still have a role to identify CAD in middle-aged sportsmen if risk factors such as cholesterol are unknown.

## Introduction

Regular physical activity is key in the prevention of cardiovascular disease, but vigorous exercise is also associated with a higher cardiovascular event risk, particularly in those with known or unknown cardiac disease [1]. Over 90% of exercise related cardiac arrests occur in men, predominantly in those aged 45 years and older, and the majority is attributable to coronary artery disease (CAD) [2, 3].

In a group of 108 recreational German marathon runners aged 50 or more years the coronary artery calcium burden was higher in marathon runners than in Framingham Risk Score (FRS) matched controls [4]. During the six years of follow up, cardiovascular events occurred in 8 participants, 7 of whom had a CACS higher than 100 Agatston units although their mean FRS placed them in a low-risk category [5]. It seems that the conventional cardiovascular risk stratification underestimates the coronary artery calcium burden in this presumably healthy cohort and that an increased awareness of a potentially higher than anticipated coronary risk is warranted [5].

These facts indicate that early identification of subclinical CAD should be an important goal in the pre-participation sports evaluation of middle-aged persons. Pre-participation screening aims to improve the safety of exercise in the fast-growing group of middle-aged sportsmen by identifying those at increased risk of cardiovascular events. Frequently used risk scores (e.g. FRS or European Society of Cardiology SCORE) tend to underestimate cardiovascular risk in these middle-aged sportsmen, as evidenced by the results of the abovementioned Marathon study [6]. The 2011 European Society of Cardiology position paper on cardiovascular evaluation of middle-aged/senior individuals engaged in leisure time sports activities advocates the use of maximal exercise testing [7]. However, although electrocardiogram (ECG) interpretation in sportsmen can be improved using a standardized ECG criteria tool [8], the low positive predictive value and high false positive rate for CAD of both resting and exercise electrocardiography in asymptomatic individuals remains a cause for concern [9, 10]. Although exercise ECG is not a standard part of routine pre-participation sports screening, it is still frequently performed in the sports medical evaluation (SME) of those aged 45 years and older.

Cardiac CT (CCT), including both non-contrast CT for CACS and contrast-enhanced coronary CT angiography (CCTA), provides direct, non-invasive visualization of the coronary arteries. Higher CACS independently predicts cardiovascular mortality, and CCTA visual [11, 12], and CCTA characterizes (calcified vs. non-calcified plaque) and quantifies the extent of the total atherosclerotic burden and severity of any coronary stenosis. However, routine CCT is not routinely performed in the SME setting because of costs, radiation exposure and the need for specific equipment and expertise. Pulse wave velocity (PWV) has recently emerged as a potential new biomarker for prediction of cardiovascular mortality independent of established risk factors such as blood pressure and cholesterol [13–15], and normal and reference values per age category based on a large European cohort have now been established [16].

In contrast to CCT, measuring arterial stiffness is easy to perform in an outpatient setting. In addition, increased arterial stiffness measured by means of PWV has been shown to be an independent predictor of cardiovascular mortality in various populations [17–21]. Numerous studies have assessed the additional value of carotid-femoral PWV for prediction of cardiovascular events in the general population [14, 22–24]. Several carotid-femoral PWV methods (SphygmoCor and Complior) are widely used to determine PWV, although both methods are observer-dependent and time-consuming [25]. The Arteriograph is a relatively new and operator-independent device, which uses oscillometric pressure curves registered by an upper arm blood pressure cuff connected to a piezo-electric sensor to determine blood pressure and PWV as validated previously [26–28]. It shows comparable results to tonometry (Complior) in healthy controls and patients with cardiovascular disease, although systematically lower values (on average 0.8 m/s lower) are found for the Arteriograph in healthy controls [29].

We set out to determine the value of PWV evaluated with the relatively new Arteriograph, in addition to routinely assessed cardiovascular risk factors (SCORE), to detect CAD on CCT in asymptomatic middle-aged sportsmen.

## Methods

### Study population

Participants were consecutively recruited from the Measuring Athlete’s Risk of Cardiovascular events (MARC) study that evaluates the additional value of CCT (both CACS and CCTA) to routine SME, including resting and exercise ECG, in asymptomatic sportsmen aged 45 years and older. Details on the MARC study design have been reported previously [7]. This study was conducted according to the principles of the Declaration of Helsinki and has been approved by the regional Medical Ethics Committee (VCMO, Nieuwegein, The Netherlands), and the local ethics committee of the University Medical Center Utrecht, The Netherlands. All participants gave written informed consent.

Asymptomatic sportsmen aged 45 years and older, without known cardiovascular disease (known coronary artery disease, MI, percutaneous coronary intervention, coronary artery bypass graft surgery, stroke, transient ischemic attack or peripheral artery disease) were included if they had undergone a SME with exercise ECG that revealed no abnormalities, according to the responsible physician. Exclusion criteria were known CAD, allergy to contrast material, and renal impairment. Both competitive and recreational sportsmen were included, with the majority of them engaged in high-dynamic high-static sports (cycling) and high-dynamic low-static sports (long distance runners).

Information regarding basic demographic data, cardiovascular risk factors (smoking status, cholesterol level, hypertension, diabetes, family history), medication use, as well as height, weight, body mass index (BMI) and blood pressure were obtained at the sports medical department. Two blood pressure measurements were obtained with a standard sphygmanometer. Total cholesterol and creatinine were measured on the day of the CT-scan if there were no recent values known within the last 6 months. Inclusion criteria for this specific study were arterial PWV measurement on the day of the CT-scan. The participants were asked to refrain from physical exercise and from consuming caffeine on the day of the CT-scan and PWV measurements.

### Definitions

CAD was defined as a CACS ≥100 AU on non-contrast CT or a ≥50% luminal stenosis on CCTA. Smoking status was classified into two levels: ever (former and current) smoker and never smoker. Hypertension was defined as a resting blood pressure above 140/90 mm Hg or the use of blood pressure lowering medication.

### Pulse wave velocity

PWV, brachial blood pressure and heart rate were measured in a supine position using a blood pressure cuff on the left arm after several minutes of rest just prior to CCT. PWV as a measure of arterial stiffness was assessed using the Arteriograph system (Tensiomed, Budapest, Hungary). One measurement was performed in each participant by one observer. The measurement was repeated in 23 (12%) cases of unsuccessful readings. Persistent unsuccessful readings did not occur. Intra-observer validation in 41 participants showed a good intra-observer agreement (Pearson’s correlation 0.9, R^2^ 0.8). One observer performed all measurements, and inter-observer validation was beyond the scope of this study. Normal values in an age-matched population were used to identify abnormal arterial stiffness measurements as a measure for subclinical atherosclerosis [15]. We assessed both a PWV cut-off using the normal value of 8.3 m/s for the population 50–59 years old (established with carotid-femoral PWV measurements) [15], as well as a PWV cut-off value of 7.5 m/s that was corrected by 0.8 m/s for the numerical lower Arteriograph PWV measurements [29]. PWV was measured as continuous data and assessed as the percentage of participants with a higher than normal PWV, based on the established cut-off value in a population with optimal blood pressure and no identified cardiovascular risk factors[16], and abovementioned cut-off value corrected for the systematically lower Arteriograph measurement.

### Cardiac CT

Image acquisition was performed with a 256-slice CT system (Brilliance iCT, Philips Healthcare). First a non-contrast prospectively ECG-triggered CCT was acquired to calculate the CACS. Scan parameters were 120 kV, 60 mAs. Images were reconstructed at a slice thickness and increment of 3 mm. CACS was quantified as Agatston scores by identifying all regions of at least 1mm^2^ with ≥130 Hounsfield units (HU) within the coronary arteries using semi-automatic software (HeartBeat-CS, Philips Healthcare) [30]. CCTA scan parameters were as follows: 120 kV; 210 mAs; 95–115 ml (depending on weight < or ≥ 80 kg) non-ionic contrast material (Ultravist 300 mg I/ml, iopromide) injected at a speed of 6–6.7 ml/s followed by 30–40 ml saline injected at the same flow rate. The CCTA was acquired with prospective ECG triggering at a mid-diastolic phase (78%). Participants with a heart rate of more than 65 beats/min received 5 to 20 mg metoprolol (Selokeen) intravenously before CCTA. All participants received sublingual nitroglycerine (nitrolingual) immediately before CCTA. Laboratory technicians performed the coronary artery calcium score using semi-automatic software. All CT scans were assessed by one of two experienced cardiac radiologists (NP, BKV), blinded for findings at the baseline assessment. The CCTA scans were assessed for obstructive stenosis (≥50%) comparing the diameters of the maximal stenosis to a reference diameter proximal and distal to the stenotic area. The PWV measurements were collected by a different reader (TLB) who was blinded for the CAC results. All relevant findings on CCT were discussed at consensus meetings with a panel of at least two (sports) cardiologists and one radiologist.

### Statistical analysis

Data are shown as mean ± SD for continuous variables where applicable, otherwise medians and 25^th^ and 75^th^ percentiles (interquartile range [IQR]) are given. All categorical data are reported as a percentage or absolute number. Student’s t test was used for differences between groups and proportions between groups were compared by means of chi-square test. Sensitivity, specificity and positive- and negative predictive values of PWV for detecting relevant CAD for both >8.3 m/s and >7.5m/s were calculated. The additional diagnostic value of PWV measurements, compared to the SCORE parameters gender, age, systolic blood pressure, total cholesterol, and ever smokers, was quantified using multivariate logistic modelling and area under the receiver operating characteristic (ROC) curve (C-statistic) analysis. Statistical significance was defined as a two-sided p-value < 0.05. The SPSS 20.0 (SPSS Inc., Chicago, IL, USA) statistical software package was used for all calculations.

## Results

Arterial PWV measurements were performed in 193 consecutive participants of the MARC cohort (MARC participants 121–313). The characteristics of these 193 middle-aged sportsmen are summarized in Table 1. Forty-seven (24%) participants were found to have CAD on CCT. Forty-three participants had a CACS ≥100 AU. The CCTA identified a total of 12 participants with luminal narrowing ≥50%, eight of whom also had a CACS ≥100 AU. Those with CAD were older (58.9 vs. 53.8 years, p<0.001), more often had hypertension (17% vs. 4%, p = 0.003), had higher total cholesterol levels (5.7 vs. 5.4 mmol/l, p<0.05) and more often were (ever) smokers (55 vs. 34% p = 0.008). Mean arterial PWV was 8.25 ± 1.9 m/s with significantly higher PWV in the participants with CAD compared to those without CAD (8.9 vs. 8.0 m/s, p = 0.02).

**Table 1:** Baseline characteristics.

Characteristic	All N = 193	CAD N = 47	No CAD N = 146	P-value ^a^
Age (years)	55.0 ± 6.6	58.9 ± 6.3	53.8 ± 6.3	<0.0001
Diabetes, n (%)	3(2)	1(2)	2(1)	0.71
Hypertension, n (%)	14 (7)	8 (17)	6(4)	0.003
BMI (kg/m2)	24.7±2.4	25.3±2.7	24.5±2.2	0.06
Ever smoked, n (%)	75(39)	26(55)	49(34)	0.008
Height (cm)	182±7.1	181±7.4	183±6.9	0.07
Weight (kg)	82±9.7	82±10.2	82±9.6	0.90
SBP (mmHg)	129±12.8	132±11.9	128±13.0	0.06
DBP (mmHg)	79±12.8	81±7.3	79±9.2	0.10
Total cholesterol (mmol/l)	5.4±0.9	5.7±0.8	5.4±0.9	0.048
PWV (m/s)	8.3±1.9	8.9±2.0	8.0±1.8	0.017
PWV >8,3 m/s, n (%)	65(34)	20(43)	45(31)	0.1
PWV >7,5 m/s, n (%)	116(60)	35(74)	81(55)	0.02
SCORE, median (IQR)	1 (1–2)	1 (1–2)	1(1–2)	0.26
Exercise tolerance (Watt)	307±47	303±46	315±51	0.2

BMI, body mass index; SBP, systolic blood pressure; DBP, diastolic blood pressure; PWV, pulse wave velocity. Data are presented as mean ± SD, proportions (%) or median values (interquartile range i.e. 25th and 75th percentile).

^a^ P-value calculated with or Pearson χ2 or Fisher’s Exact Test (two sided) where appropriate, between cad and no cad groups.

For the PWV cut-off value >8.3m/s the sensitivity to detect CAD was 43%, specificity 69%, positive predictive value 31% and negative predictive value was 79%. For the PWV cut-off value >7.5m/s the sensitivity to detect CAD was 74%, specificity 45%, positive predictive value 30% and negative predictive value was 84%. In univariate analysis, age, (ever) smoking and PWV were significantly associated with CAD with respective odds ratios (OR) of 1.15 per year (95% CI 1.08–1.21, p<0.001), 1.62 (95% CI 1.2–2.3, p = 0.006) and 2.3 per m/s (1.1–4.9, p = 0.01). The predictive capacity of PWV in relation to relevant CAD was characterized by a C-statistic of 0.60 (95% CI 0.50–0.69, p = 0.050). The area under the curve (AUC) was 0.78 (95% CI = 0.709 to 0.848) for the SCORE parameters gender, age, systolic blood pressure, total cholesterol, and ever smokers. Adding PWV measurements to the SCORE model did not appreciably change the AUC (model 2, AUC 0.78 95% CI 0.710–0.848, p = 0.99) indicating limited additional diagnostic value of PWV assessed with a relatively new technique to SCORE parameters.

## Discussion

In this study performed in asymptomatic sportsmen aged 45 years or older, we found that the sensitivity of Arteriograph derived PWV measurements for detecting subclinical CAD was only fair when using a cut-off of 8.3 m/s, and fairly good for 7,5 m/s with a sensitivity of 74%.

When information on established risk factors (SCORE) was available, the additional value of PWV appeared to be limited. However, as cholesterol is not always known, one might still consider implementing arterial PWV measurements with the Arteriograph in the routine SME of middle-aged sportsmen. The diagnostic sensitivity of PWV in identifying those with a high risk of CAD is promising as 3 in 4 asymptomatic participants with subclinical CAD can be identified. This may help justify referral of asymptomatic sportsmen for CCT based on available risk factors and PWV measurements.

Early identification of CAD should be an important goal in the pre-participation sports evaluation of middle-aged persons. While sudden cardiac death in younger athletes (35 years or younger) is mainly caused by cardiomyopathies, electrical heart disease and coronary anomalies, in older individuals it is predominantly caused by CAD (80%) [3, 31]. A recent paper on cardiac arrest during long distance running implicated a causal role for demand ischemia in athletes with (unknown) CAD [32]. Absence of coronary plaque rupture in these persons was surprising because prior data [33] and expert consensus documents [31] have suggested that exercise-induced acute coronary events result from atherosclerotic plaque disruption and coronary thrombosis. This means that screening for relevant CAD is perhaps more important than only performing exercise testing for a hemodynamically significant coronary stenosis. However coronary calcium can only be regarded as a proxy measure for coronary artery disease. Although it is unlikely to find someone with extensive coronary atherosclerosis who has no CAC [34], persons with a CACS <100 AU can still suffer from a myocardial infarction [35, 36]. Although CAD has been considered the primary cause of exercise related SCD in individuals >35 years, there is evidence that diseases traditionally associated with SCD in young athletes also play a significant role in this population [32, 37, 38]. Therefore, only screening for CAD may not detect all middle-aged and older individuals at risk of SCD.

Our study is the first to evaluate the potential role of PWV to identify subclinical CAD in asymptomatic middle-aged sportsmen that have undergone a routine SME. There is substantial interest in refining cardiovascular risk prediction to better address preventative therapy among those individuals considered to be at low or moderate risk according to current guidelines [39]. Therefore, additional cardiovascular biomarkers have been identified, including the mean common carotid intima-media thickness, which has been shown to have clear additional value in prediction of CV events on top of the FRS and ESC SCORE [40]. Although PWV may have potential as a biomarker [13–15], it remains unclear whether PWV measurements have additional clinical value in the daily practice of screening middle-aged sportsmen. Since the established risk scores tend to underestimate cardiovascular risk in middle-aged sportsmen, as demonstrated by Mohlenkamp et al. [6], we investigated whether aortic stiffness, as measured by PWV, improved identification of sportsmen with subclinical atherosclerosis that warrants further testing. However, this study has been critiqued for a higher proportion of smokers in the self-selected study group. Our results support the association between established risk factors (including smoking), arterial stiffness and CAD on CCT in middle-aged sportsmen. Our results are comparable to a recent meta-analysis including more than 17.000 participants [21]. The authors stated that aortic PWV has value beyond conventional risk factors to predict mortality and future cardiovascular events for younger individuals at intermediate risk (improved risk prediction of 13%). Although it was still predictive for older individuals (hazard ratio 1.23 for a subgroup above 70 years of age), the addition of PWV to the adjusted cardiovascular prediction models only increased the C-statistics to a modest degree, suggesting that PWV did not add much to standard risk assessment. A possible explanation is that systolic blood pressure is a better surrogate of aortic stiffness in older people than in younger people and the authors concluded that including PWV in models already containing systolic pressure might limit the predictive value [21]. Furthermore another recent publication that was not included in the meta-analysis showed comparable results for risk prediction of CAD with PWV in a large cohort of elderly subjects [41]. The authors concluded that aortic stiffness measurement in addition to FRS resulted in only limited reclassification rates and stated that although aortic stiffness is associated with risk of CAD in elderly free of known CAD it provides no additional value in cardiovascular risk stratification.

Our study must be interpreted within the context of its limitations. First of all, we performed measurements in a relatively small group of 193 participants. Although the number of consecutive participants was considerable for a study of its kind, it remains a relatively small study of selected men (all were Caucasian with at least college education). The fact that they consented to participate after undergoing a SME may have led to additional selection bias (e.g. those with a higher chance of cardiovascular disease because of a positive family history). In order to avoid possible selection bias we compared the baseline criteria of the MARC population to a large group (n = 725) of male recreational sportsmen aged ≥45 years who recently underwent a SME in the southern region of the Netherlands (Maxima Medical Centre, Veldhoven). The groups were similar in age (mean age 55.0 vs. 54.3 years) systolic blood pressure (129 vs. 129 mmHg), body mass index (24.7 vs. 25.0 kg/m^2^), smoking status (current 3.1 vs. 5.1%), and fitness level (workload 314 vs. 309 Watt), indicating that our group is representative for sportsmen that undergo a SME. Remarkably the consecutive participants who underwent additional PWV had a slightly higher incidence of CAD compared to the whole cohort (24 vs. 18%, p = 0.09). However, this difference was not significant (P = 0.09) and the consecutive participants who underwent additional PWV were similar in age (54.7 vs. 54.9 m/s, P = 0.4) SCORE (both 1 with IQR 1–2), systolic blood pressure (SBP 127 vs. 129 mmHg P = 0.9), body mass index (BMI 24.9 vs. 24.7 kg/m^2^ P = 0.7) smoking status (ever smoking 37% vs 39% P = 0.8) and fitness level (workload 314 vs. 307 Watt P = 0.3).

Second, the Arteriograph is a relatively new device for the assessment of PWV. Although it has been validated in some studies, [26, 29, 42], to our knowledge no prospective outcome studies have been carried out with this device. Ideally, we should have performed a confirmatory study to compare our Arteriograph PWV measurements to the standard carotid-femoral PWV method, however this was beyond the scope of this study. Therefore, before the Arteriograph can be implemented, we should acquire more clinical evidence that the Arteriograph can provide additional prognostic value to the traditional CAD risk measurements, particularly in older sportsmen who have a higher risk of CAD related events.

Third, we used established cut-off values for a population with optimal or normal blood pressure and no identified CV risk factors. It is possible that the normal range however may be different in our specific (trained) population. A recent study demonstrated that high intensity aerobic interval training has the ability to reduce arterial stiffness in a cohort of treated hypertensive women after 16 weeks of follow-up [43]. However this effect has neither been demonstrated for moderate-intensity continuous exercise training nor has this been demonstrated in an asymptomatic male cohort free of known cardiovascular disease. Of note, the majority of our participants are engaged in moderate intensity continuous exercise training (cycling, long distance running).

In summary our study demonstrates that although limited additional value was found for PWV evaluated with the Arteriograph on top of established risk factors to identify CAD, PWV measurements may still have a role in the routine SME of middle-aged sportsmen to help identify those at a higher risk for relevant CAD. Larger studies of asymptomatic sportsmen in the SME setting are required to verify that functional arterial measurements can have added clinical value for cardiovascular risk stratification.

## Supporting Information

S1 Dataset Raw data. (ZIP)
